# Divergent proliferation patterns of distinct human hair follicle epithelial progenitor niches *in situ* and their differential responsiveness to prostaglandin D2

**DOI:** 10.1038/s41598-017-15038-9

**Published:** 2017-11-09

**Authors:** Talveen S. Purba, Michael Peake, Bessam Farjo, Nilofer Farjo, Ranjit K. Bhogal, Gail Jenkins, Ralf Paus

**Affiliations:** 1 Centre for Dermatology Research, School of Biological Sciences, University of Manchester, MAHSC and NIHR Biomedical Research Centre, Manchester, UK; 2 MRes Programme, Department of Biological Sciences, University of Huddersfield, Huddersfield, UK; 3 Farjo Hair Institute, Manchester, UK; 4 Unilever R&D Colworth, Bedfordshire, UK

## Abstract

Human scalp hair follicles (hHF) harbour several epithelial stem (eHFSC) and progenitor cell sub-populations organised into spatially distinct niches. However, the constitutive cell cycle activity of these niches remains to be characterized *in situ*. Therefore, the current study has studied these characteristics of keratin 15+ (K15), CD200+ or CD34+ cells within anagen VI hHFs by immunohistomorphometry, using Ki-67 and 5-ethynyl-2′-deoxyuridine (EdU). We quantitatively demonstrate *in situ* the relative cell cycle inactivity of the CD200+/K15+ bulge compared to other non-bulge CD34+ and K15+ progenitor compartments and found that in each recognized eHFSC/progenitor niche, proliferation associates negatively with eHFSC-marker expression. Furthermore, we also show how prostaglandin D2 (PGD2), which is upregulated in balding scalp, differentially impacts on the proliferation of distinct eHFSC populations. Namely, 24 h organ-cultured hHFs treated with PGD2 displayed reduced Ki-67 expression and EdU incorporation in bulge resident K15+ cells, but not in supra/proximal bulb outer root sheath K15+ progenitors. This study emphasises clear differences between the cell cycle behaviour of spatially distinct stem/progenitor cell niches in the hHF, and demonstrates a possible link between PGD2 and perturbed proliferation dynamics in epithelial stem cells.

## Introduction

The hair follicle (HF) houses multiple epithelial stem (eHFSC) and progenitor cell populations in human^1–13^ and murine skin^14–18^. In the human HF, below the well-defined ‘bulge’ niche at the level of the arrector pili muscle attachment site, the outer root sheath (ORS) also contains a minimum of two additional progenitor populations that differentially express eHFSC-associated markers^1-11^.

Namely, in human anagen VI HFs, the bulge niche can be identified by markers CD200, keratin 15 (K15) and keratin 19 (K19) (with overlapping but unique expression patterns in this region^7,10,11^ (Figs S1 and S2)). Additional compartments include the sub-bulge, marked by basal layer CD34 expression as well as heightened SOX9 immunoreactivity, and sitting below this (just above the hair matrix) is the supra/proximal bulb ORS compartment (pbORS), which can be identified as a second K15+ and K19+ rich population, absent of prominent epithelial CD200/CD34 expression^1-11^.

Accurately distinguishing between these ORS stem/progenitor compartments and further characterising them is critical to assess the physiological effects of intervention in translational hair research. This is important given that these regions are anatomically distinct, with likely unique and specific functions (especially with regards to their varying proximity to different skin signalling centres such as the dermal papilla (DP) or HF associated adipose tissue^19–21^). For example, the dynamics of cells comprising these regions could have differing functions during the HF cycle e.g. during anagen maintenance or the anagen-catagen transition^3,10,11,22^.

Within the epithelium of skin and its appendages, proliferation of somatic stem cells is required to maintain homeostasis^10,23^, be it for differentiation, self-replenishment or repair. So far, current studies have only qualitatively examined overall keratinocyte proliferation in the human HF^3,10,24–27^, or have characterised isolated human eHFSC populations via *in vitro* colony forming efficiency assays or FACS analyses^1,2,6,10,28,29^.

Whilst FACS and other *in vitro* quantitative methods can provide valuable and instructive data, these often fall short without complementary quantitative *in situ* data, which is dependent on the knowledge of the exact localisation of analysed cells of interest. This can be of critical importance for data interpretation, especially in the human HF. This can also be problematic when given skin (stem) cell populations have a shared marker expression profile despite being unique in their function and localisation. (i.e. K15+ cells in the epithelium of both human skin and hair^7^).

Moreover, no previous work has yet performed a systematic comparative and quantitative *in situ* analysis of proliferation in epithelial stem/progenitor cells and their immediately adjacent progeny on tissue sections of human anagen HFs. This is an important, yet unobvious, gap in the literature needed as an instructive reference point for future studies and to aid the reliable determination of regions of interest for cell cycle analyses, either during experimental investigation or when studying HFs in pathological conditions.

We have addressed this gap by analysing proliferating cells (via Ki-67 expression) and cells undergoing DNA synthesis (via 5-ethynyl-2′-deoxyuridine (EdU) incorporation)^25,30,31^ within distinct epithelial stem/progenitor cell compartments of the ORS in combination with cells the eHFSC markers K15, K19, CD200 and CD34^10^.

In addition, to showcase the relevance of performing proliferation analyses in distinct progenitor cell compartments and sub-populations directly on HF tissue sections, we examined the effects of Prostaglandin D2 (PGD2) and its non-enzymatic metabolite, 15-deoxy-Δ12,14-prostaglandin J2 (15d-PGJ2), on Ki-67 expression and EdU incorporation in epithelial HF stem/progenitor cells via *ex vivo* human HF organ culture experiments.

PGD2 was selected as a candidate modulator of eHFSC proliferation dynamics for several reasons. Firstly, androgenetic alopecia (AGA) reportedly shows a loss of CD200+/CD34+ cells whilst retaining a K15+ cell population^1^. These changes in stem/progenitor cells may be linked to PGD2, given that AGA scalp skin was shown to have upregulated lipocalin-type prostaglandin D2 synthase (L-PGDS) and PGD2^32^. Furthermore, PGD2 and 15d-PGJ2 have been reported to inhibit human HF growth *ex vivo* ^32–34^. Therefore, we assessed whether PGD2 and 15d-PGJ2 modulate the cell cycle dynamics of human eHFSCs and progenitor cells *in situ*.

## Results

### Distinct human eHFSC/progenitor cell-containing compartments differ strikingly in their cell cycle dynamics

**Figure 1:**
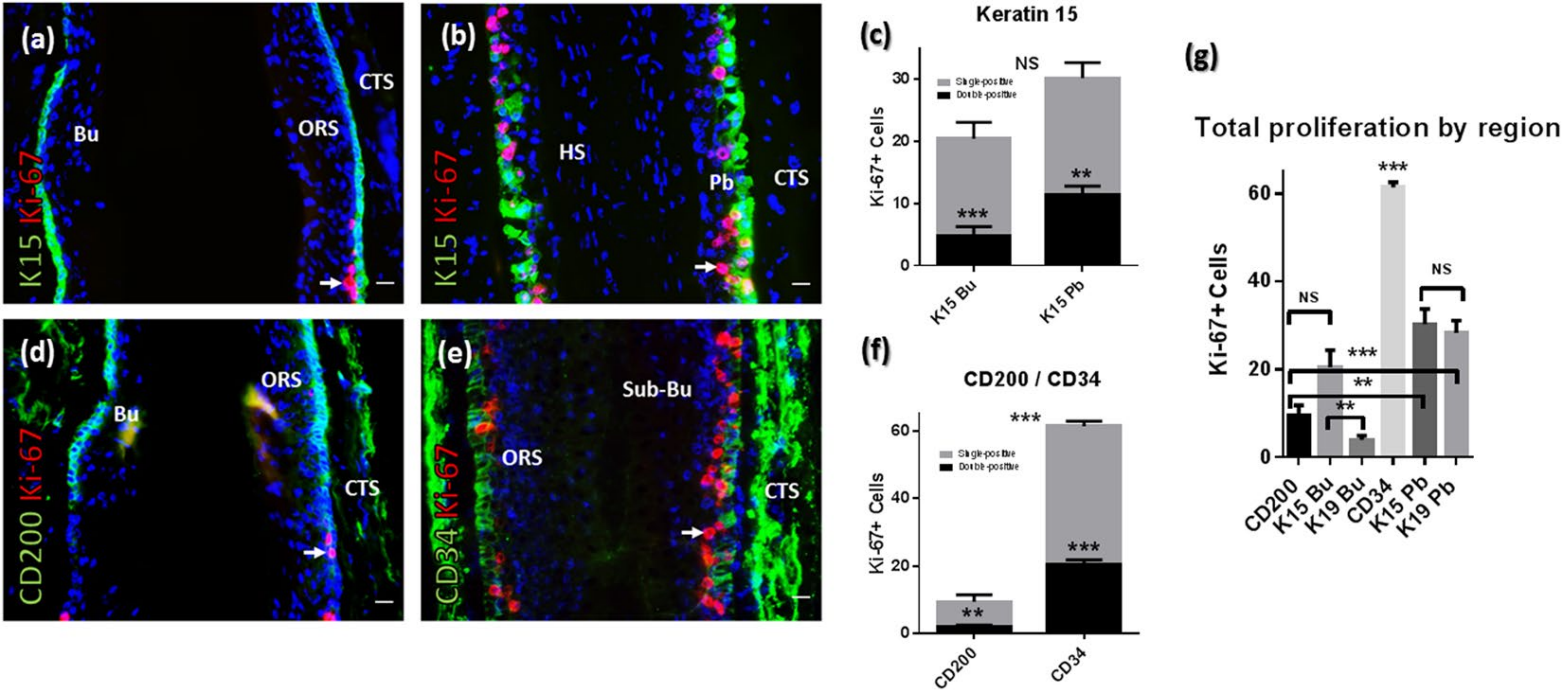
Distinct eHFSC/progenitor containing ORS populations differ characteristically in proliferation and quiescence. (**a**,**b**) Ki-67 immunofluorescence with K15 in the bulge (**a**) and pbORS (**b**). (**c**) K15+ cells show fewer Ki-67+ cells versus their (K15−) progeny in both compartments (paired t-tests). Total Ki-67+ cells is not different between the K15+ bulge and pbORS (unpaired t-test). (**d**,**e**) Ki-67 immunofluorescence with CD200 (**d**) and CD34 (**e**) in the bulge and sub bulge. (**f**) CD200+ and CD34+ cells show less Ki-67 expression versus their progeny (paired t-test and Wilcoxon test respectively). There are more cycling cells in the CD34+ region compared to the CD200+ region (Mann Whitney U test). (**g**) One way ANOVA (***) with multiple comparisons; the CD34+ region harbours the most Ki-67+ cells. Proliferation is greater in the K15+ bulge versus the K19+ bulge. Proliferation is lower in the CD200+ bulge versus the K15+ and K19+ pbORS, and in the K19+ bulge compared to the K15+ pbORS (**) (not shown). Proliferation is not different between bulge region CD200 and K19 compartments (not shown). See Fig. S3 for further K19 data. Analyses conducted using mean values per HF analysed: n = 9–15 HFs (Patient N = 4–5). **P = <0.01; ***P = <0.001. Significance asterisks *within* bars denote double vs. single comparisons. Significance asterisks *between* bars in chart denote comparisons of total Ki-67+ counts. Arrows indicate proliferating cells in the HF epithelium. 20 µm scale bars. Error bars are standard error. Bu – Bulge; CTS – connective tissue sheath; HS - hair shaft ORS; outer root sheath; Pb – pb(proximal bulb)ORS.

First, we assessed cell cycle activity through G1-S-G2-M via analysis of Ki-67 expression in relation to K15+ (bulge, pbORS), K19+ (bulge, pbORS), CD200+ (bulge) and CD34+ (sub-bulge) cell compartments of male human occipital scalp HFs in anagen VI^10,11,25^ (Figs 1, S1 and S2) through the performance and analysis of double-immunofluorescence stains on tissue sections. This revealed that the total number of Ki-67+ cells did not differ significantly between the K15+ bulge and K15+ pbORS regions, despite a trending increase in the latter compartment (Fig. 1a–c). On the other hand, when both K19+ compartments were compared and examined, significantly more Ki-67+ cells were detected in the pbORS compared to the bulge (Fig. S3a–c). The discrepancy between these K15 and K19 bulge versus pbORS proliferation analyses may reflect their distinct, yet overlapping, expression patterns in the human HF epithelium^7,10,11^ (Fig. S2). The CD200+ bulge region^1,2,10,35^ showed significantly fewer Ki-67+ cells *in situ* when compared to the CD34+ sub-bulge (Fig. 1d–f). Importantly, all eHFSC/progenitor populations (CD200+, CD34+, K15+ and K19+) showed significantly less Ki-67 positivity than their single-positive (i.e. CD34−/Ki-67+) progeny in the suprabasal ORS (Figs 1c,f and S3c).

Performing a comparative analysis of the total number of Ki-67+ cells between all eHFSC/progenitor cell compartments clearly demonstrated that the CD34+ sub-bulge contained the highest number of proliferating cells compared to any other examined stem/progenitor cell region in human HFs (Fig. 1g). The number of Ki-67+ cells in the CD200 zone of the HF was significantly lower in comparison to all other regions except the K15+ (despite trending differences) and K19+ demarcated bulge regions. However, there was a significant difference in the number of Ki-67+ cells between the K15+ and K19+ bulge regions, but their counterpart pbORS populations were not different from one another in this regard (Fig. 1g).

We then performed EdU incorporation experiments through incubation of EdU during *ex vivo* HF organ culture (see methods). EdU labelling allows the selective visualisation of DNA synthesis, to verify actual cell proliferation i.e. progression beyond the G1 restriction point, which cannot be defined by Ki-67 alone^25,31^.

**Figure 2:**
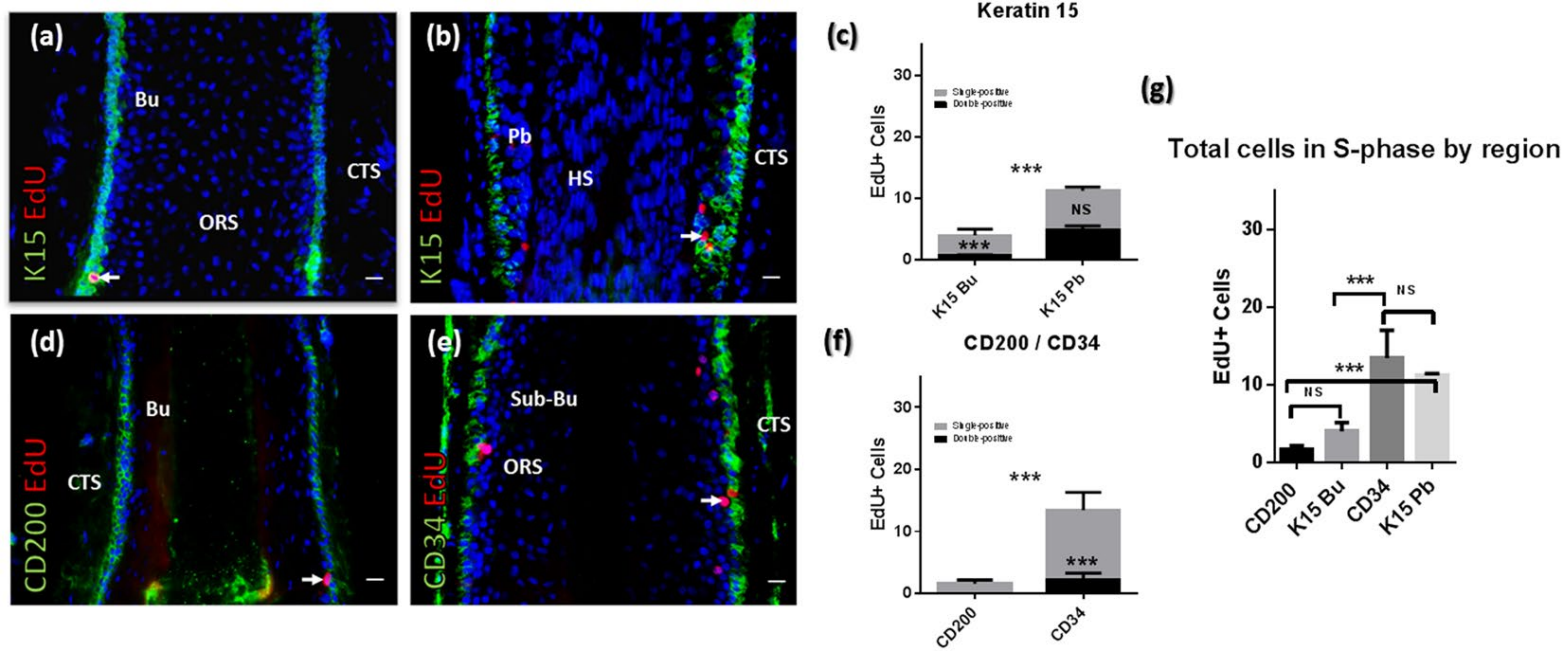
Distinct eHFSC/progenitor containing ORS populations differ in their patterns of EdU incorporation (DNA synthesis). (**a**,**b**) EdU incorporation with K15 immunofluorescence in the bulge (**a**) and pbORS (**b**). (**c**) Bulge K15+ cells, but not pbORS K15+ cells, show less EdU incorporation versus their K15- progeny (Wilcoxon and paired t-tests respectively). Total number of EdU+ cells is lower in the K15+ bulge region versus the K15+ pbORS region (Mann Whitney U test). (**d**,**e**) EdU incorporation with CD200 (**d**) and CD34 (**e**) in the bulge and sub bulge. (**f**) CD200+ bulge stem cells show no DNA synthesis (EdU incorporation) (in sample analysed), whilst their suprabasal progeny show limited DNA synthesis. CD34+ cells show less EdU+ cells versus their CD34- progeny (Wilcoxon test). There are more EdU+ cells in the CD34+ region versus the CD200+ region (unpaired t-test). (**g**) One way ANOVA (***) with multiple comparisons; the CD34+ region shows greater EdU+ cells versus the K15+ bulge region (and CD200 region (***) as seen in Fig. 2f), but is not different from the K15+ pbORS. The K15+ pbORS region showed more EdU+ cells than the CD200 region (and K15+ bulge (*) as also demonstrated in Fig. 2c). Analyses conducted using mean values per HF analysed: n = 9–14 HFs (Patient N = 5). *P = <0.05; ***P = <0.001. Significance asterisks *within* bars denote double vs. single comparisons. Significance asterisks *between* bars in chart denote comparisons of total EdU+ counts. Arrows indicate proliferating cells in the HF epithelium. 20 µm scale bars. Error bars are standard error. Bu – Bulge (quiescent epithelial stem cell zone); CTS – connective tissue sheath; HS- hair shaft ORS; outer root sheath; Pb – pb(proximal bulb)ORS.

Through fluorescent click-chemistry based detection and analysis of EdU incorporation in eHFSC/progenitor compartments^25^ on HF tissue sections, it was found that patterns of DNA synthesis mirrored Ki-67 expression patterns (Figs 2 and S2). Differences between the K15+ bulge and the K15+ pbORS was in this instance significant (in contrast to when Ki-67 was analysed in the same K15+ compartments), whereby the K15+ bulge region demonstrated relatively fewer cells in S-phase when compared to the K15+ pbORS (Fig. 2a–c). Furthermore, similar to Ki-67 data, the total number of cells undergoing DNA synthesis was markedly heightened within the CD34+ sub-bulge region when compared to the CD200+ bulge region (Fig. 2d–f). Notably, analysis of EdU+ incorporation within the CD200+ compartment highlighted a complete lack of detectable DNA synthesis in these cells within the sample set analysed (albeit this does not preclude it). This, considered alongside similar Ki-67 data, establishes the CD200+ region being largely constituted by a veritable ‘zone of quiescence’ under normal conditions.

Again, consistent with the Ki-67 data described above, eHFSC/progenitor marker-expressing cells also showed significantly less EdU incorporation than their single-positive (i.e. CD34−/EdU+) suprabasal progeny, except in the K15+ pbORS (Fig. 2c,f). Notably, the number of detected EdU+ cells (S-phase) was far fewer than the number of Ki-67+ cells within the same eHFSC/progenitor cell regions (given Ki-67 is expressed throughout all active cell cycle phases including S-phase) (Figs 2 and S2).

The total number of EdU+ cells was significantly higher in the CD34+ sub-bulge and K15+ pbORS regions when compared to K15+/CD200+ bulge region (Fig. 2g). The number of EdU+ cells was not significantly different between the CD200+ bulge and K15+ bulge regions (Fig. 2g). However data showed that on average there was greater, albeit variable (thus non-significant), numbers of proliferating cells in the K15+ versus CD200+ bulge (Figs 1g and 2g). This can be attributed to differing intra-bulge region expression patterns (Figs S1 and S2) whereby K15 expression extends beyond the ‘zone of quiescence’ (as demarcated by a lack of Ki-67+ and EdU+ cells under normal conditions), whereas CD200 does not (Fig. S2). Finally, there was no difference in the total number of EdU+ cells between the CD34+ regions versus the K15+ pbORS (Fig. 2g), in contrast to Ki-67 data (Fig. 1g).

These *in situ* analyses on HF tissue sections sheds new light by building a complex picture of the proliferation dynamics of the multiple ORS eHFSC/progenitor sub-populations and their immediate progeny in the human anagen HF. They support the concept that the human bulge, like its murine counterpart^14–18^, is a zone of relative eHFSC quiescence, in line with previous FACS-based data^1,6^. This contrasts with the relatively proliferative sub-bulge/pbORS progenitor cell compartments, which in themselves also show differences from one another, affirming their uniqueness both spatially, phenotypically and functionally. Strikingly, these data also document how proliferation in ORS compartments (i.e. bulge/sub-bulge/pbORS) is negatively associated with eHFSC/progenitor marker protein expression, strongly suggesting that down-regulation of stemness markers coincides with cell cycle activity.

### Proliferation in distinct human eHFSC/progenitor cell sub-populations is differentially affected by PGD2 stimulation

PGD2 is upregulated in balding scalp and inhibits hair growth *ex vivo* ^32^, which could be linked to reported changes in eHFSC populations in hair loss^1^. We therefore applied cell cycle analyses *in situ* to determine whether proliferation within distinct stem/progenitor sub-populations that express defined eHFSC- markers is affected by treatment with PGD2 or its non-enzymatic metabolite, 15d-PGJ2.

**Figure 3:**
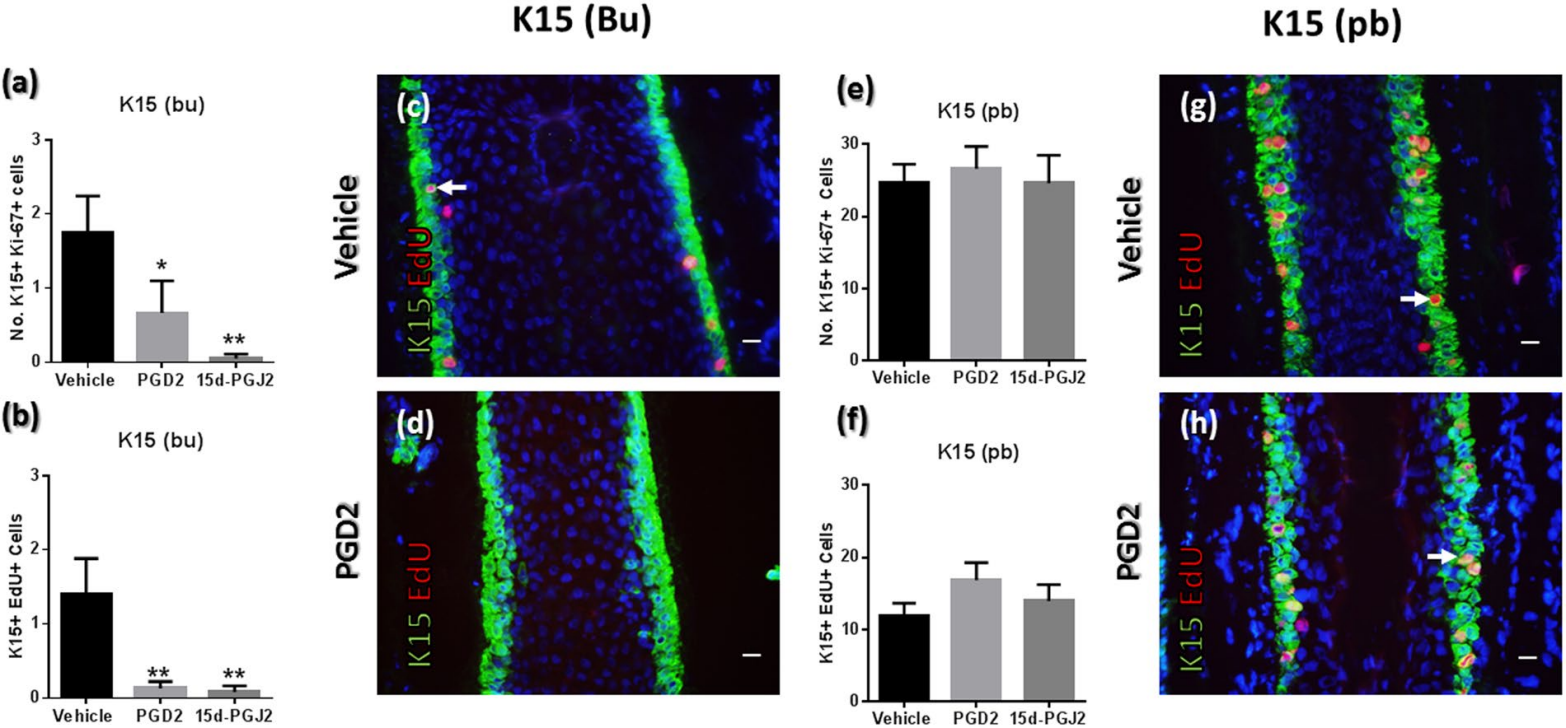
PGD2 and 15d-PGJ2 treatment reduces K15+ cell cycle activity in bulge eHFSCs but not in pbORS progenitors. (**a**,**b**) The number of Ki-67+ (**a**) and EdU+ (**b**) cells is reduced within K15+ bulge cells by PGD2 and 15d-PGJ2 treatment. (**c**,**d**) Representative images demonstrating inhibition of proliferation (EdU incorporation (DNA synthesis/S-phase) shown) in the K15+ bulge cell by PGD2 (Mann Whitney U tests). Analyses conducted using mean values per HF analysed: n = 9–15 HFs (Patient N = 4–6). *P = <0.05; **P = <0.01. (**e**,**f**) The number of Ki-67+ and EdU+ cells in K15+ pbORS cells is unaffected by PGD2 (**e**) and 15d-PGJ2 (f) treatment (unpaired t-tests). (**g**,**h**) Representative images demonstrating that proliferation in K15+ pbORS cells is unaffected by PGD2 treatment. (EdU incorporation shown). Analyses conducted using mean values per HF analysed: n = 8–16 HFs (Patient N = 4–6). Errors bars are standard error. Arrows indicate proliferating cells in the HF epithelium. Scale bars are 20 µm. Bu – Bulge; Pb – pb(proximal bulb)ORS. All comparative analyses represent number of double (i.e. K15+ Ki-67+) positive cells. Data from 24 h HF organ culture experiments.

Following 24 h *ex vivo* HF organ culture experiments (see methods) we first found, via quantitative immunohistomorphometry, that PGD2 and 15d-PGJ2 both suppressed the number of proliferating K15+ bulge cells (reduced number of both K15+ Ki-67+ and K15+ EdU+ cells in this compartment) following treatment (Fig. 3a–d). In striking contrast, treatment with these prostaglandins did not affect proliferation within K15+ pbORS region progenitor cells (Fig. 3e–h). These contrasting results reinforce the independent and semi-autonomous nature of these distinct K15+ cell populations.

**Figure 4:**
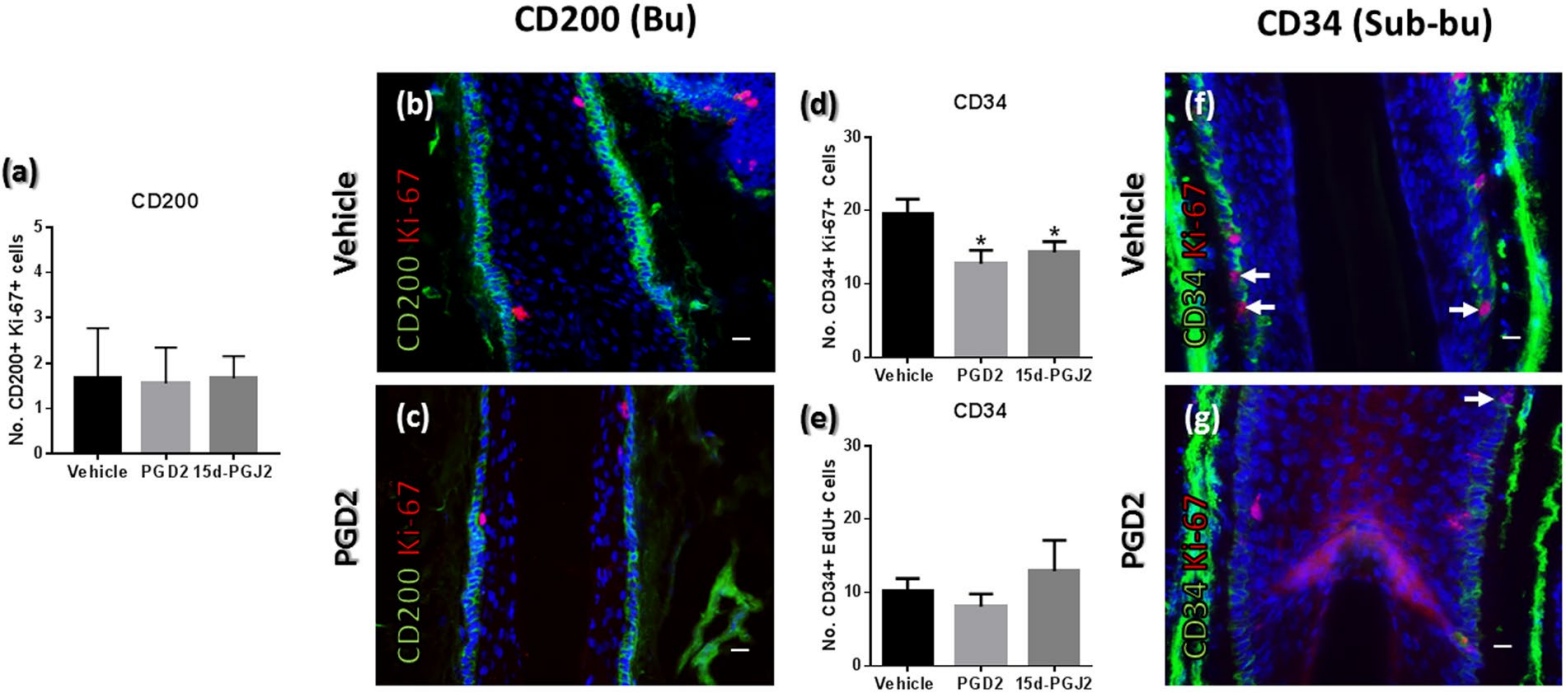
Effects of PGD2 and 15d-PGJ2 treatment on CD200+ and CD34+ progenitor cell proliferation. (**a**) PGD2 and 15d-PGJ2 treatment does not affect proliferation in CD200+ cells of the bulge (Mann Whitney U tests) (**b**,**c**) CD200/Ki-67 immunofluorescence in vehicle (**b**) and PGD2 treated (**c**) bulge region. Note that CD200+ cells did not show EdU incorporation in initial analyses, see Fig. 2f. n = 7–11 HFs (Patient N = 3–4). (**d**,**e**) The number of CD34+ Ki-67+ (**d**) cells is reduced in the sub-bulge ORS by PGD2 and 15d-PGJ2, but the number of CD34+ cells undergoing S-phase (CD34+ EdU+) was not affected (**e**). Unpaired t tests performed (with exception of vehicle vs. 15d-PGJ2 CD34 analysis; Mann Whitney U test). (**f**,**g**) Representative images of reduced Ki-67 expression in CD34+ cells following PGD2 treatment. n = 8–15 HFs (Patient N = 3–5). *P = <0.05. Arrows indicate proliferating CD34+ cells. Analyses conducted using mean values per HF analysed. Errors bars are standard error. Scale bars are 20 µm. All comparative analyses represent number of double positive cells (i.e. CD34+ Ki-67+). Data from 24 h HF organ culture experiments.

Treatment with PGD2 and 15d-PGJ2 did not significantly affect the number of CD200+ Ki-67+ cells (Fig. 4a–c). This is in contrast to the effects of these prostaglandins in the K15+ bulge, which underscores their status as independent sub-populations^1,5,6,10,35^ (Figs 1, S1 and S2) with differing characteristics, fitting prior observations^1^. Notably, serial sections showed that cells most prominently expressing K15 are seen to reside below the CD200+ population (Fig. S1).

PGD2 and 15d-PGJ2 both reduced the number of CD34+ Ki-67+ cells, but this was not met with a change in the number of CD34+ EdU+ cells in this compartment (Fig. 4d–g). This implies arrest induced by PGD2 (and 15d-PGJ2) in G1, whereby the total number of Ki-67+ cells is reduced^36^ whereas the already cycling S-phase fraction of cells remain unaffected, as has been observed previously^37^.

Notably, in all progenitor cell marker defined compartments, the effects of PGD2 and 15d-PGJ2 were consistent with one another (Figs 3 and 4). Players in the PGD2 pathway have been described to exert anti-proliferative/cell cycle perturbing effects^37–45^, which aligns with the above described effects within responsive ORS compartments.

Further probing the prominent effects of PGD2 treatment within the K15+ bulge population, we found that the number of (late G2/) mitotic (phospho histone H3+ Ser10 (pH3+)) bulge (and pbORS) K15+ cells did not significantly change in response to PGD2 treatment (Fig. S4). When compared to data from Ki-67 and EdU analyses, this suggests limited sensitivity of pH3 as a metric for proliferation in eHFSCs due to the short duration of M-phase in mammalian cells^46^, compounded by relatively low cell turnover in this region.

We then investigated the effects of PGD2 treatment following extended (96 h) *ex vivo* organ culture experiments. We found that the cell cycle inhibitory effect of PGD2 treatment was no longer significant in bulge K15+ cells (and still unchanged in the pbORS) (Fig. S5), despite decreasing trends. Here, bulge K15+ stem cells in both groups showed massively uncharacteristic increases in proliferation. This is reminiscent of already perturbed proliferation in eHFSCs analysed at 24 h (Figs 3 and 4). This indicates potential caveats of extended *ex vivo* HF organ culture for studying *in situ* eHFSC/progenitor cell proliferation dynamics, given that their behaviour can deviate from homeostatic conditions.

## Discussion

The current and first quantitative *in situ* study on human HF tissue sections shows that distinct eHFSC and progenitor cell niches in male human occipital scalp anagen VI HFs^1–13^ are not only spatially, but functionally distinct, given their profound differences in Ki-67 expression and EdU incorporation.

Our study adds much-needed yet previously missing tissue context to prior analyses that were based on FACS^1,6^, and goes further by quantitatively characterising proliferation in the human HF in several unique, precisely localised progenitor cell populations spanning at-least 3 anatomically distinct compartments. This type of quantitative immunohistomorphometry analysis has been lacking in previous studies.

Namely we demonstrate *in situ* that the bulge region, containing and demarcated by CD200+/K15+ (and K19+) eHFSCs, shows low cell cycle activity. Relative to these bulge region populations, both the CD34+ sub-bulge and K15+ (and K19+) pbORS regions display greater overall proliferation, with intrinsically differing patterns between them (i.e. CD34+ sub-bulge vs. K15+ pbORS Ki-67 expression). These latter compartments constitute parts of the ‘non-permanent’ portion of the HF epithelium, which is remodelled and renewed during HF cycling^47^, whereby epithelial CD34 expression is limited to anagen^48^.

The hierarchy between eHFSC markers remains mysterious, e.g. identifying a ‘true’ stem cell population (which may not concretely exist, given context specific plasticity). We instead posit that, at least in the bulge, eHFSCs represent a heterogeneous and dynamic pool (Figs S1 and S2), likely varying greatly in three dimensions^7,49,50^. This cell pool can be recognised histologically by a band of nuclei within the basal layer with clearly limited Ki-67 expression/EdU uptake in the bulge region (Fig S2).

Importantly, we further demonstrate that throughout the ORS, eHFSC-associated markers (K15, K19, CD200 and CD34) show down-regulation with cell cycle activity, not demonstrated before on human HF tissue sections via quantitative analyses.

We also describe novel *in situ* data on how treatment of human HFs with PGD2 and its non-enzymatic metabolite 15d-PGJ2 associates with differential effects on proliferation within distinct eHFSC/progenitor cell populations within the anagen HF epithelium. These experiments were performed in response to findings presented by Garza *et al*. who had shown an upregulation of L-PGDS and PGD2 in AGA scalp^32,34^. In another study, Garza *et al*., also described the loss of CD200+ and CD34+ cells, whilst reporting that a K15+ cell population is maintained^1^.

Namely, we show the inhibition of proliferation in select eHFSC/progenitor sub-populations (K15+ bulge and CD34+ sub-bulge) by PGD2 and 15d-PGJ2, whilst leaving other sub-populations (CD200+ bulge and K15+ pbORS) unaffected. This reinforces not only anatomical, but functional differences of distinct compartments of the HF ORS with regards to their constitutive cell cycle behaviour.

These effects in responsive cell populations are consistent with prior findings that describe the relationship between the PGD2 pathway and cell cycle inhibitory effects in cell lines^37–45^. Albeit why different progenitor populations/regions respond differently to PGD2/15d-PGJ2 (or are maintained in AGA), i.e. selective cell cycle perturbations in a region spanning the anagen K15+ bulge and CD34+ sub-bulge, is not yet clear.

These cell cycle observations seemingly contrast with what has been reported in AGA scalp i.e. loss of CD200+/CD34+ populations and retention of K15+ populations^1^ (via FACs analysis on interfollicular AGA scalp skin, which may have contained K15+ basal epidermal keratinocytes^51^). However our data could imply that possible defects in the ability of K15+ bulge stem cells to convert to CD200+/CD34+ progenitor cells, as previously conjectured in AGA^1^, is linked to cell cycle-dependent impairments in differentiation via PGD2.

However, our experiments notably utilised anagen VI occipital scalp HFs that are not inherently susceptible to miniaturisation in male pattern baldness and may not be immediately comparable to HFs from AGA scalp skin. Furthermore, miniaturisation is a slowly progressing process occurring within a dynamically cyclic mini-organ. Therefore, despite the instructiveness of HF organ culture, this makes it difficult to completely mimic the chronic effects of elevated PGD2 on epithelial stem/progenitor cell behaviour as well as the complex cellular pathogenesis of AGA in a short term *ex vivo* model. Indeed, prolonged culture of HFs outside of their skin environment may also disrupt homeostatic epithelial stem cell proliferation dynamics as indicated by our observations.

To close, this work further promotes the future examination of cell cycle dynamics and read-out parameters in human HF biology *in situ* ^25,30^ via *ex vivo* organ culture experiments, be this via further investigation of the PGD2 pathway^34^, other related signalling systems e.g. PPARy^52^, or during clinical testing of candidate anti-hair loss agents. Basic *in situ* characterisation of cell cycle behaviour, including via additional parameters i.e. cell cycle-regulatory proteins^31,53^, is also of interest in other contexts such as stem cells in the wound response^54^, cancer^55^ and in the study of hair pathology, e.g. lichen planopilaris^35^. Our study underscores the instructiveness of human HF organ culture^30^ as an excellent, clinically relevant model system for studying the cell cycle dynamics of adult human stem cells under complex epithelial-mesenchymal interaction conditions and within their physiological tissue habitat.

## Materials and Methods

### Tissue handling

Occipital scalp skin was received from the Farjo Hair Institute, Manchester, UK. This tissue, obtained via follicular unit extraction or strip harvesting, was donated by adult male patients undergoing hair transplantation surgery, under informed consent and institutional ethical approval granted by the University of Manchester ethics committee. All experiments were conducted in accordance with relevant University of Manchester policies and guidelines.

Tissue was obtained, processed and stored abiding to the Human Tissue Act (2004), UK. Full length anagen VI human HFs were microdissected and were either isolated immediately (e.g. all Ki-67 analyses were conducted on freshly microdissected anagen VI HFs from male occipital scalp) or subject to *ex vivo* HF organ culture (see below)^25,30^. Dissected HFs (aligned side by side) were frozen in liquid nitrogen within Cryomatrix™ (#9990422, Thermo Fisher Scientific, Waltham MA) and stored at −80 °C. Vertical cryosections of HFs were subsequently prepared at a thickness of 7 µm onto Superfrost™ Plus slides (#4951PLUS4, Thermo Fisher Scientific).

### PGD2 and 15d-PGJ2 human HF organ culture

Human HFs were cultured serum-free *ex vivo* ^30^ in the presence of 10 µM PGD2 (#sc-201221, Santa Cruz) or 5 µM 15d-PGJ2^32^ (#sc-201262, Santa Cruz) or vehicle (dimethyl sulfoxide) for 24 h or 96 h. For 96 h culture experiments, media was changed daily. HF serum free media consists of Williams E Medium (#12551-032 500 ml, Thermo Fisher Scientific), supplemented with 100 U/ml Penicillin and 100 μg/ml Streptomycin (#P4333-100 ML, Sigma, St Louis MO), 2 mM L-glutamine (#G7513-100 ML, Sigma), 10 μg/ml insulin (#I9278-5 ML, Sigma) and 10 ng/ml hydrocortisone (#H4001 Sigma). Following culture HFs were then processed as above.

### Immunofluorescence

Immunofluorescence procedure was adapted from protocol as described previously^11^, whereby frozen tissue sections were immersed and fixed in ice-cold Acetone for 10 minutes and incubated overnight with primary antibody at 4 °C, then incubated with fluourescent secondary antibody for 45 minutes at room temperature. Cell nuceli were stained with 4′,6-diamidino-2-phenylindole (DAPI). See Table S1 for dilutions and details of primary and secondary antibodies employed.

### EdU incorporation

EdU incorporation was carried as described previously^31^, where HFs were incubated in 20 µM EdU for 4 hours during HF organ culture prior to isolation. Tissue was processed as above, and fluorescent detection of EdU incorporation on tissue sections was achieved via a copper-catalysed click reaction as per the manufacturer’s instructions (Table S1). Double-staining was achieved by prior performance of immunofluorescence protocol prior to EdU detection.

### Quantitative immunohistomorphometry and analysis

In this study, our choice of markers to identify epithelial stem/progenitor cells for quantitative immunohistomorphometry^11,12,25,30^ (alongside cell cycle markers e.g. Ki-67, EdU) was decided based upon their capacity to consistently and reliably mark specific and distinct regions of the ORS at the basal layer in (fresh isolated) human HFs, as defined in previous studies. Indeed eHFSC markers chosen for analysis were thus purposely limited to cell surface markers or cytokeratins.

Namely: (**a**) CD200 labels an epithelial cell population within the bulge^1,2,5,6,10,35^. (**b**) CD34 labels cells below the CD200+ bulge in the sub-bulge, and is downregulated again in the proximal bulb ORS^4,5,6,9,10,48^. (**c**) K15 (labelled via LHK15 clone) shows strongest expression in the bulge compartment, is reduced in the sub-bulge yet becomes stronger again in the pbORS region.^5,10,11,28^. (**d**) K19 labels the bulge, becomes intermittent in the sub-bulge and increases again in the pbORS, and shows expression that is similar yet distinct from K15^3,5,7,9–11^.

Moreover, Ki-67 and EdU identify proliferating cells with great specificity, allowing straightforward analysis, especially where the fluorescent detection system of the latter is reliant on specific click-chemistry based methodology (see above).

To facilitate accurate quantitation, it was vital to further ensure: (**a**) Preparation of optimal tissue sections of anagen VI human hair follicles. (**b**) Usage of carefully established and reproducible immunostaining protocols. (**c**) Performance of experiments in parallel. (**d**) Consistency in image acquisition (i.e. exposure/zoom). (**e**) Usage of stringently defined reference areas.

The number of positive cells, averaged per HF from repeat tissue sections, was manually quantified using ImageJ software (NIH, Bethesda, MA) ‘Fiji’ version, via use of inclusive ‘cell counter’ plugin. ORS region of interest was defined by specific basal stem/progenitor cell expression (see above), in areas where marker signal was most intense within the field of view. Repeat and independent morphometric analyses were performed by distinct investigators (which yielded highly comparable results), as to ensure robustness and reproducibility in our reported counts. Analyses were conducted using HFs from 4–6 patients (see also legends for n). Statistical analysis (e.g. Mann Whitney U test, unpaired t-test, one way ANOVA), was performed within GraphPad Prism (GraphPad Software, La Jolla, CA). Choice of statistical test was selected appropriately, considering normality testing and variance via D’Agostino-Pearson omnibus test and F test respectively. Paired analyses (paired t-test, Wilcoxon signed-rank test) were performed to compare double-versus single positive cells within the same region of the same HF. However, paired analyses between distinct ORS niches were not performed due to an inability to often obtain, from a single HF, overlapping data that completely spanned each marker and region of interest that would be required for such a comparison.

## Electronic supplementary material

Supplementary Information
