# Supplementary material for: Divergent proliferation patterns of distinct human hair follicle epithelial progenitor niches in situ and their differential responsiveness to prostaglandin D2
Source: Sci Rep. 2017 Nov 9;7:15197. doi: 10.1038/s41598-017-15038-9 (PMC5680340; doi:10.1038/s41598-017-15038-9)
Supplement: Supplementary file 1 — Supplementary Information [file 41598_2017_15038_MOESM1_ESM.pdf]

Supplementary materials to:

Divergent proliferation patterns of distinct human hair follicle epithelial progenitor niches *in situ* and their differential responsiveness to prostaglandin D2

Talveen S Purba, Michael Peake, Bessam Farjo, Nilofer Farjo, Ranjit Bhogal, Gail Jenkins, Ralf Paus*

Antibody/ assay	Host	Localisation	Dilution/ Concentration	Catalogue	Vendor
**Alexa Fluor 488/594**	Goat anti mouse/rabbit	-	1:200	A11001, A11005, A11008, A11037	Thermo Fisher Scientific
**CD200**	Mouse	Bulge	1:100	MCA1960GA	AbD Serotec / Bio-Rad
**CD34 (My10)**	Mouse	Sub-bulge	1:10	347660	BD Biosciences
**EdU incorporation**	-	-	20 µM	C10339	Thermo Fisher Scientific
**Keratin 15 (LHK15)**	Mouse	Bulge & pbORS	1:200	AB1385	Abcam
**Keratin 19**	Mouse	Bulge & pbORS	Pre-diluted	AB87000	Abcam
**Ki-67**	Rabbit	-	1:50	AB16667	Abcam

Table S1 Antibodies used for immunofluorescence

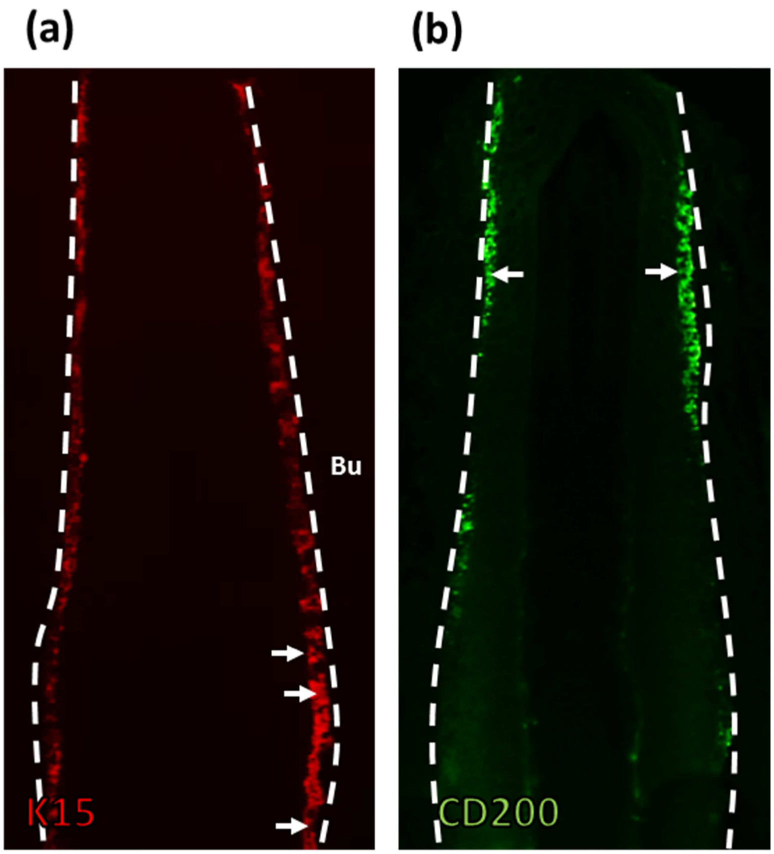

Figure S1 Co-localisation of CD200 and K15 expression within the human HF bulge

Serial sections highlighting that CD200 and K15 signal co-localise to the bulge (Bu), where the most prominent expression of these markers localises to the upper and lower bulge respectively (arrows). Note that CD200 labels a more restricted subset of cells. Images taken at 200x and subsequently merged using Keyence image analysis software.

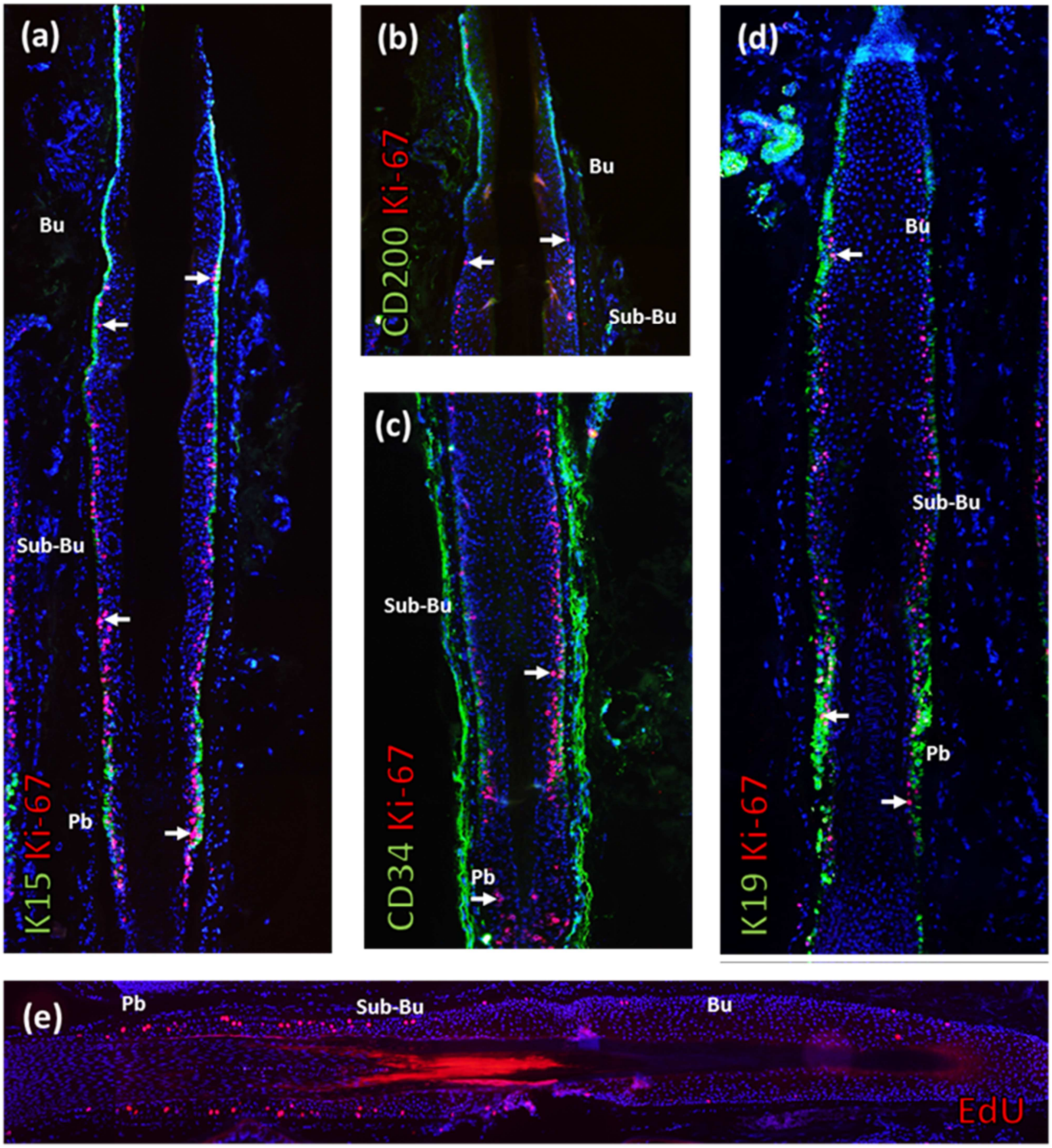

Figure S2 Localisation of stem/progenitor markers and proliferation in the human HF ORS

(a-d) Ki-67 double immunofluourescence with K15 (a), CD200 (b), CD34 (c) and K19 (d). CD200 marks the bulge (Bu), a zone of minimal Ki-67 expression, whereas K15 marks this zone too, but extends beyond it, coinciding with an increase in the total number of Ki-67+ cells. K15 (and K19, where expression is intermittent) is then downregulated in the sub-

bulge (Sub-Bu), within a region where CD34 expression increases, and vice versa again in the proximal bulb (Pb) ORS (pbORS) compartment. Arrows indicate proliferating cells in the HF epithelium. Note that expression of K15/K19 co-localise but show distinct expression patterns, as described previously [7,10,11] suggesting that they represent functionally distinct progenitor cell populations. Images taken at 200x and merged using Keyence image analysis software.

(e) Localisation of S-phase+ cells in the ORS resembles the distribution of Ki-67 cells, with limited DNA synthesis within the bulge compared to the sub-bulge and pbORS under normal conditions. Arrows indicate proliferating cells in the HF epithelium. Regions are approximate. Pb – pb(proximal bulb)ORS.

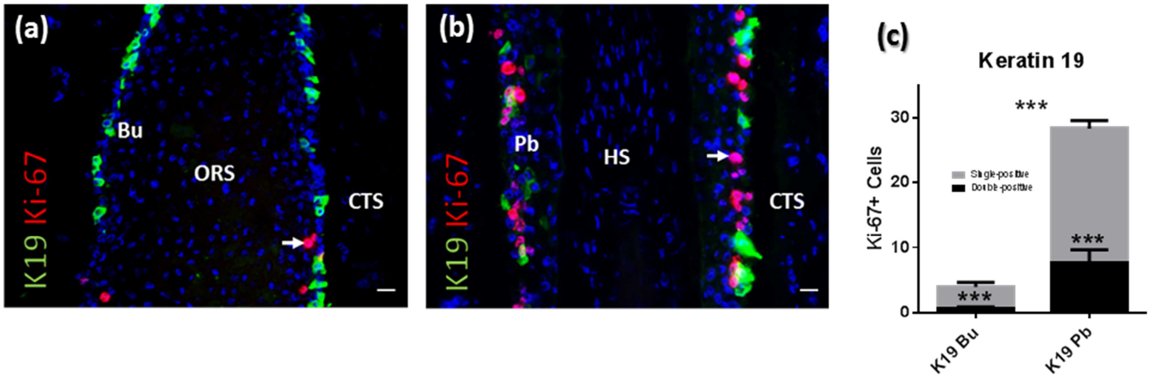

Figure S3 Patterns of proliferation in K19+ stem/progenitor populations

a-c) Total number of Ki-67+ cells is significantly greater within the K19+ pbORS region compared to K19+ bulge region (Mann Whitney U test). Both the bulge and pbORS show significantly fewer double positive cells (K19+Ki-67+) compared to single positive cells (K19-Ki-67+) (paired t-tests). Patient N=6 (total 12 HFs analysed per region). P=<0.001 (***).

Significance asterisks *within* bars denote double vs. single comparisons. Significance asterisks *between* bars in chart denote comparisons of total Ki-67+ counts. Arrows indicate proliferating cells in the HF epithelium. 20 µm scale bars. Error bars are standard error. Bu – Bulge; CTS – connective tissue sheath; HS - hair shaft ORS; outer root sheath; Pb – pb(proximal bulb)ORS

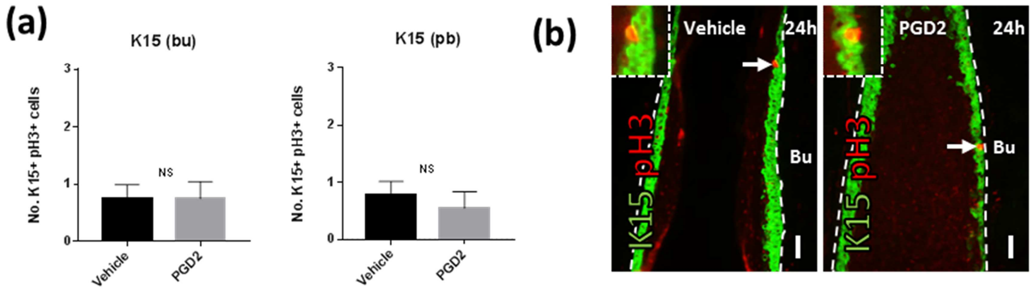

Figure S4 The number of K15+ pH3+ cells remains constant following PGD2 treatment

(a) Number of K15+/phospho histone H3 (pH3)+ double-positive cells in bulge and pbORS is unaffected by 24h PGD2 treatment (Mann Whitney U tests) **(b)** Representative images of pH3 bulge expression K15+ cells in control and treated HFs . Patient N=3; Bu - 10 HFs per group, Pb - 7-8 HFs per group. Scale bars are 10 µm. Arrows point to positive signal in the HF epithelium.

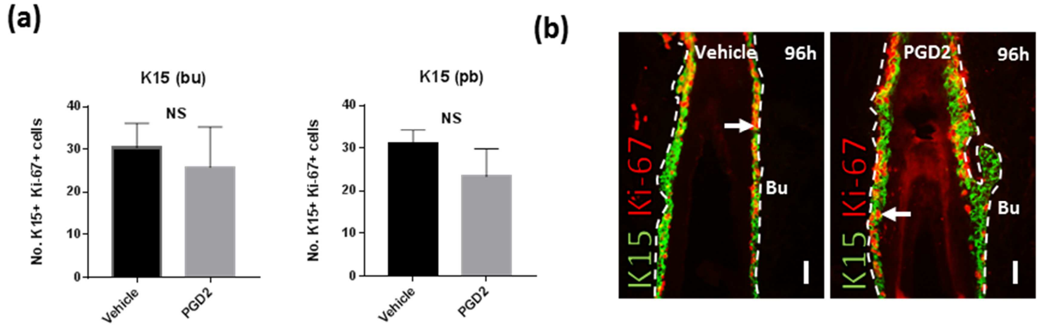

Figure S5 Effects of 96 h PGD2 culture on K15+ cell proliferation

(a) 96h HF organ culture in the presence of 10µM PGD2 (administered daily) did not significantly alter the number of bulge or pbORS K15+ Ki-67+ double positive cells compared to vehicle. Instead, extended organ culture resulted in the hyper proliferation of this otherwise quiescent compartment. Patient N=2; Bu - 6-8 HFs per group; pbORS - 5-10 HFs per group (Mann Whitney U tests). Scale bars are 10 µm. Positive cell number analyses conducted using mean values per HF analysed. Arrows point to positive signal in the HF epithelium.
